# Accurate extraction of WSe2 FETs parameters by using pulsed I-V method at various temperatures

**DOI:** 10.1186/s40580-016-0091-9

**Published:** 2016-11-21

**Authors:** Sung Tae Lee, In Tak Cho, Won Mook Kang, Byung Gook Park, Jong-Ho Lee

**Affiliations:** Department of Electrical and Computer Engineering, Seoul National University, Seoul, 151-747 South Korea

**Keywords:** Two-dimensional materials, WSe_2_ FETs, Pulsed *I*-*V* method, DC method, Temperature

## Abstract

This work investigates the intrinsic characteristics of multilayer WSe_2_ field effect transistors (FETs) by analysing Pulsed *I*-*V* (PIV) and DC characteristics measured at various temperatures. In DC measurement, unwanted charge trapping due to the gate bias stress results in *I*-*V* curves different from the intrinsic characteristic. However, PIV reduces the effect of gate bias stress so that intrinsic characteristic of WSe_2_ FETs is obtained. The parameters such as hysteresis, field effect mobility (μ_eff_), subthreshold slope (*SS*), and threshold voltage (*V*_th_) measured by PIV are significantly different from those obtained by DC measurement. In PIV results, the hysteresis is considerably reduced compared with DC measurement, because the charge trapping effect is significantly reduced. With increasing temperature, the field effect mobility (μ_eff_) and subthreshold swing (*SS*) are deteriorated, and threshold voltage (*V*_th_) decreases.

## Background

Two-dimensional(2D) layered materials such as graphene and boron nitride offer new opportunities in the field of electronics with its excellent physical, chemical properties [1–6]. Though graphene has been studied most widely, its lack of bandgap limits its application. Transition metal dichalcogenides (TMDCs) provide a solution to this problem with their sizable bandgap energy and ultra-thin form of layers. Among them, WSe_2_ FETs can be very appealing for the nanoscale electronic applications and blackplanes for flat panel display (FPD) due to their high mobility (~100 cm^2^/V·s), excellent on/off ratio(~10^7^), and low subthreshold swing (SS, ~70 mV/decade) [7–9].

When we simulate the circuits which consist of WSe_2_ FETs, it is necessary to know the parameters of the FETs. Most of the parameters reported up to now were extracted from the *I*-*V* characteristics obtained by the DC method. However, these values were not correct, because a large hysteresis is observed due to the gate bias stress during the DC measurement [10–12]. On the other hand, in the Pulsed *I*-*V* (PIV) measurement, the effect of gate bias stress is reduced greatly so that intrinsic characteristic of WSe_2_ FETs can be obtained [13, 14]. Thus PIV method is considered as a reasonable method for estimating the performance and reliability of semiconductor devices. The purpose of PIV method is to avoid the negative effects such as self-heating and transient trapped charges. Therefore, the PIV method can provide the accurate device parameters needed for improved computer-aided-engineering (CAE) software models. However, there have been no reports on the parameter extraction from the fabricated WSe_2_ FETs at various temperatures. In this work, we show the *I*-*V* curves measured by PIV and DC measurement methods, and compare the parameters extracted from the results obtained by both methods at various temperatures (−30 to 40 °C).

## Experimental details

**Fig. 1:**
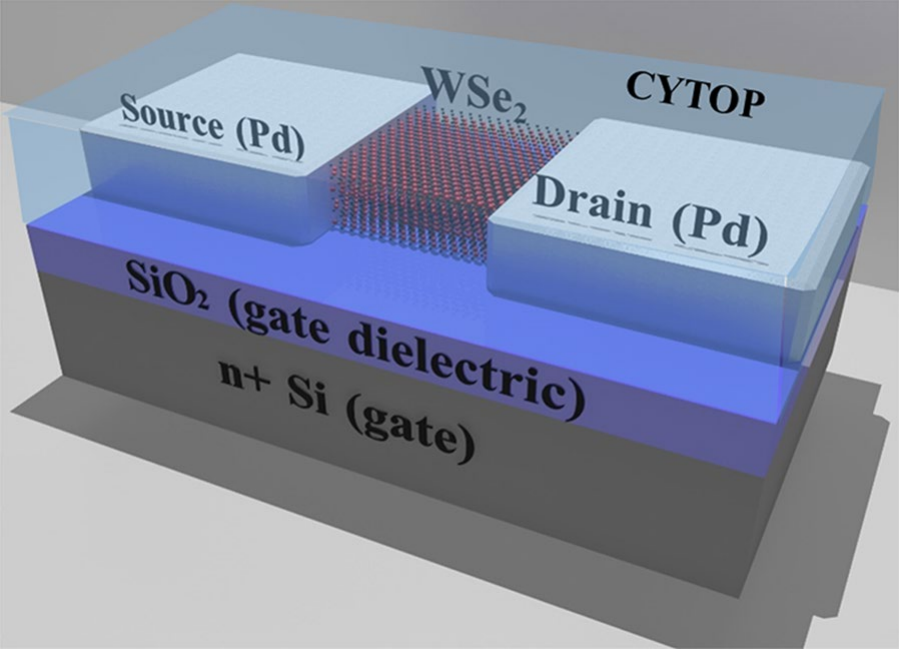
Schematic diagram of multi-layer WSe_2_ FETs with a heavily *n*-doped Si bottom-gate and Pd source/drain contact

Figure 1 shows the perspective view of the structure of a multilayer WSe_2_ FET with the bottom gate structure. An *n*-type silicon wafer which was heavily doped (ρ ~ 0.005 Ω·cm) by phosphorus is used as a starting substrate and also plays a role as back-gate electrodes. After thermal oxidation in dry oxygen at 950 °C, 35 nm thick thermal oxide which serves as gate insulator was grown on the heavily doped Si wafer. Then, WSe_2_ flakes were mechanically exfoliated from bulk WSe_2_ crystals and transferred on SiO_2_/Si substrate by using a polydimethylsiloxane (PDMS) stamp. The multi-layer WSe_2_ flakes on the substrate were annealed at 350 °C for 2 h in the ambient of a mixed gas of argon and hydrogen. Photolithographic patterning and electron beam evaporation of Pd (~70 nm), followed by lift-off in acetone, create source and drain electrodes on the WSe_2_ flakes with a good Ohmic contact. *However, one of the key limitations for TMDC devices, like other types of low-dimensional material, comes from the intrinsic nature of instability associated with easy adsorption of gaseous molecules such as oxygen and moisture due to the large surface areas of low-dimensional materials. Absorbed gaseous molecules and moisture can act as a charge trap site* [15]. *To prevent the molecules being absorbed, we adopted flourinated polymer (CYTOP; CTL-809* M*, Asahi Glass Co., Ltd) passivation so that the drift of the drain current in MoS*_*2*_ *FETs was reduced greatly* [16]. Therefore, the backside of the WSe_2_ flake was encapsulated by the CYTOP with typical spin coating process. Then, after thermal evaporation of SiOx(~50 nm) on the CYTOP, for a surface promoter layer during PR coating, pad opening was completed by dry etching(~SF_6_/CF_4_) via PR patterns.

## Result and discussion

**Fig. 2:**
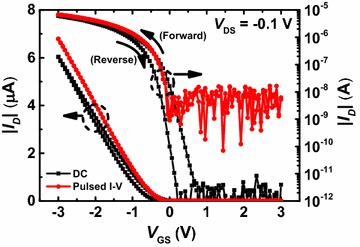
Comparison of *I*_D_-*V*_GS_ characteristics of a WSe_2_ FET measured by DC method and PIV (*t*_on_ = 10^−4^ s, *t*_off_ = 1 s, *V*_base_ = 0 V) methods at 20 °C

**Fig. 3:**
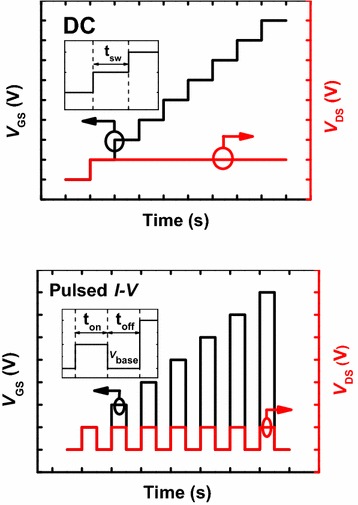
Explanation of the measurement methods for (**a**) DC and (**b**) PIV methods. The insert in **a** explains the step width at each bias step (*t*_sw_). The *t*_on_, *t*_off_, and *V*_base_ in the inset of **b** are 10^−4^ s, 1 s, and 0 V, respectively. In PIV, the drain bias is synchronized with the gate bias

Figure 2 shows the transfer curves (*I*_D_–*V*_GS_) measured at 20 °C from the FET with a *W/L* of 30/10 μm at a drain-to-source voltage (*V*_DS_) of −0.1 V. We carried out the measurement using WGFMU (Waveform Generator and Fast Measurement Unit) module installed in an Agilent B1500 semiconductor parameter analyser. *In DC and PIV measurement, the V*_*GS*_ *is scanned from 3 to −3* V *(forward) and then from −3 to 3* V *(reverse).* The transfer curves obtained by DC measurement show a large hysteresis. Because of the *V*_GS_ stress during the DC measurement, the charges can be trapped or de-trapped at the WSe_2_/SiO_2_ interface and/or the backside of WSe_2_ flakes, therefore the threshold voltage (*V*_th_) can be shifted positively or negatively. In Fig. 2, we can clearly observe the hysteresis in DC *I*_D_–*V*_GS_ curves represented by square symbols, but not in the PIV curves. The drain currents measured by the DC method are smaller than those measured by the PIV method when the *V*_GS_ is larger than *V*_th_ in magnitude. If we extract the carrier mobility from the DC *I*_D_–*V*_GS_ curves, the mobility seems to be degraded. Since some of the trapped charges stay trapped until the gate polarity is switched in the DC measurement, the large hysteresis is observed in DC measurement [17]. Note the off–current obtained by the PIV method is much higher than that obtained by the DC method in Fig. 2 because the low limit of the WGFMU module in measurement current is ~10^−8^ A. In PIV measurement, we investigated the optimized condition to reduce gate bias stress as small as possible. Figure 3a, b depict bias scheme for the DC and Pulsed *I*-*V* measurements, respectively. The *t*_sw_ in the inset of Fig. 3a represents the step width at each bias step. The drain bias (*V*_DS_) is fixed at −0.1 V from the start of the measurement. The *t*_on_, *t*_off_, and *V*_base_ in the inset of Fig. 3b stand for turn-on pulse width (10^−4^ s), turn-off pulse width (1 s), and the bias during turn-off (0 V), respectively [17]. *During the period, both rise and fall times are 10* ^*−7*^s. The *V*_GS_ pulse during *t*_off_ is set to 0 V to minimize the *V*_GS_ stress effect. Shorter *t*_on_ and longer *t*_off_ are necessary to suppress the effect of the trapping and detrapping of the charges. Note the drain bias is synchronized with the gate bias and the bias is −0.1 V during *t*_on_.

**Fig. 4:**
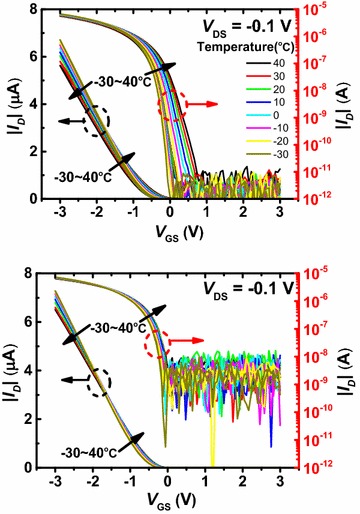
**a** Transfer curves of multilayer WSe_2_ FET measured by DC method using the bias scheme shown in Fig. 3a at various temperatures (−30 to 40 °C). **b** Transfer curves of multilayer WSe_2_ FET measured by PIV method using the bias scheme shown in Fig. 3b at various temperatures (−30 to 40 °C)

Figure 4a, b show the transfer curves measured by DC and PIV methods, respectively, at various temperatures at a *V*_DS_ of −0.1 V. As mentioned in Fig. 2, the hysteresis is much suppressed by measuring the device with the PIV method. Mobility (*μ*_eff_), subthreshold swing (*SS*), *V*_th_ are extracted from the transfer curves at various temperatures.

**Fig. 5:**
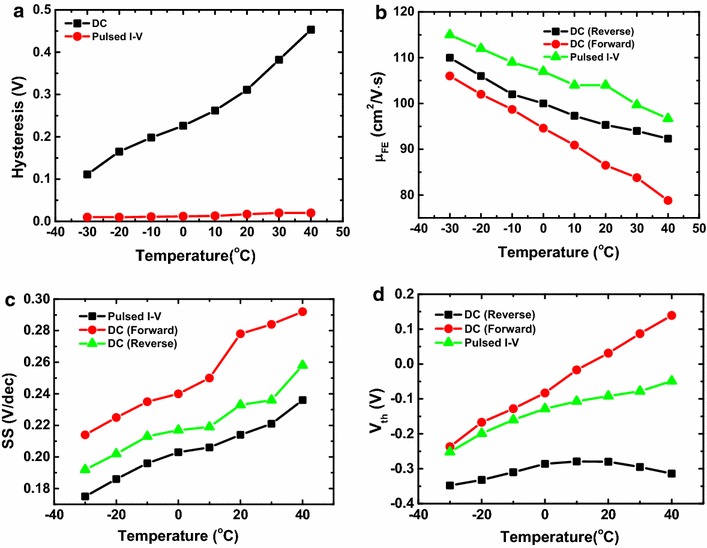
Temperature dependency of **a** hysteresis, **b** field effect mobility, **c** subthreshold swing, **d** threshold voltage obtained from multilayer WSe_2_ FET. All parameters are extracted from transfer curves

In Fig. 5, shown is temperature dependency of electrical parameters extracted from transfer curves of multilayer WSe_2_ FETs. In Fig. 5a, the hysteresis is clearly observed in the *I*_D_–*V*_GS_ curves obtained by DC method, but ignorable hysteresis in the curves measured by PIV method. The hysteresis was defined as the *V*_GS_ difference at a fixed drain current of 10^−8^ A between the forward and reverse scans. Electrons or holes during the measurement of the DC method can be trapped or detrapped into the traps at the interface due to *V*_GS_ stress, which leads to a positive or negative shift in the *V*_th_. As the temperature increases, the hysteresis in the curves measured by DC method increases because elevated temperature activates the trapping and detrapping process of carriers. It seems that the time constants for the carrier trapping and detrapping in given temperature range are longer than the *t*_on_ (10^−4^ s) used in this work so that the increase of hysteresis during PIV method can be ignorable. The variation of the hysteresis with the *t*_on_ was studied in [17].

Figure 5b shows the temperature dependency of mobility. The field effect mobility was extracted from the maximum point of transconductance (*g*_m_) using the following equation

1 $$\mu_{eff} = \frac{{Lg_{m} }}{{WC_{i} V_{DS} }}$$

where C and *g*_m_ are gate capacitance per unit area and the transconductance, respectively. The *μ*_eff_ was deteriorated as the temperature increases from −30 to 40 °C. Phonon scattering is enhanced with rising temperature, which leads to the degradation in the carrier mobility. According to the relation of *μ*_*eff*_ ∝ *T* ^*−γ*^, the *γ*s for the forward and reverse scans are 1.149 and 0.682, respectively. However, the decrease rate obtained by the PIV is 0.646. The mobility measured by PIV method is greater than the mobility measured by DC method, because in the DC measurement hole was trapped and it decreases the current, which decreases apparently the effective mobility. However, in Pulsed *I*-*V* measurement, the trapped hole was detrapped during *t*_off_ and the mobility was not degraded appreciably. Besides, mobility measured in DC forward scan is lower than the mobility measured in DC reverse scan. The hole density in the channel is reduced due to the charge (hole) trapping during DC forward scan, so the slope of *I*-*V* curves is reduced, which results in reduced mobility. During DC reverse scan, the discharging is mainly occurred by hole emission to the valance band. The holes need some energy to be emitted to the valence band. Therefore, the traps do not discharge significantly until the gate bias is below threshold voltage (*V*_th_), so the carrier density in the channel is reduced.

In addition, as the temperature rises, the difference between the mobility measured by DC forward and reverse scans increases as shown in Fig. 5b. The reason for the difference is explained as follows. Compared to the case of the forward scan, the holes in the reverse scan are increasingly emitted to the valence band with increasing temperature since increasing temperature increases the energy of the holes trapped.

Figure 5c shows subthreshold swing (*SS*) measured at various temperatures. The *SS* was defined as *V*_GS1_(*V*_GS_ at *I*_*d*_ = 10^−9^ A)–*V*_GS2_(*V*_GS_ at *I*_*d*_ = 10^−8^ A). As temperature increases, the *SS* increases as given by

2 $$SS = 2.3\frac{kT}{q}\left( {1 + \frac{{C_{dm} }}{{C_{ox} }}} \right) .$$

*SS* measured by DC measurement is greater than *SS* measured by PIV, because charge trapping decreases the current in DC measurement which makes *SS* larger. Furthermore, *SS* measured by DC forward scan is larger than the *SS* measured by DC reverse scan, because as explained above the slope of DC forward scan is smaller than that of DC reverse scan. The difference between *SS* measured by DC forward scan and *SS* measured by DC reverse scan increases as temperature rises. This phenomenon can be explained by the physics explained in the mobility difference with the temperature.

The threshold voltage (*V*_th_) was calculated by reading a gate-source voltage (*V*_GS_) at a constant current (*I*_d_ = 10^−8^ A). In PIV method, the threshold voltage shifts to the positive direction due to the increase of thermally activated carrier density. At any temperature, the threshold voltage measured by DC forward scan is in the rightmost position, and the threshold voltage measured by DC reverse scan is in the leftmost position. The reason can be explained as follows. During DC forward scan, the electron was trapped before *V*_GS_ reaches *V*_th_ so the threshold voltage moves to the right. On the other hand, during DC reverse scan, the hole was trapped before *V*_GS_ reaches *V*_th_ so the threshold voltage moves to the left. Furthermore, the difference between *V*_th_s measured by DC forward and reverse scans increases with increasing temperature, since the hysteresis increases.

## Conclusions

In this paper, we extracted key parameters of WSe_2_ FETs accurately at various temperatures by adopting pulsed *I*-*V* (PIV) method. The behavior of these parameters are different from that of the parameters obtained by DC method. For example, the decrease rates of the hole mobility are 1.149 and 0.646, respectively, for DC (forward scan) and PIV methods. By using PIV method, we could obtain accurate behavior of hysteresis, hole mobility, subthreshold swing, and threshold voltage with increasing temperature. We observed notable degradation of parameter with increasing temperature. The mobility is degraded, subthreshold swing increases and threshold voltage moves to the right as temperature rises. The parameters obtained by using PIV method are accurate and will be useful in the simulation of WSe_2_ FETs circuit at various temperatures.
